# Nitric Oxide and Strigolactone Alleviate Mercury-Induced Oxidative Stress in *Lens culinaris* L. by Modulating Glyoxalase and Antioxidant Defense System

**DOI:** 10.3390/plants12091894

**Published:** 2023-05-05

**Authors:** Riti Thapar Kapoor, Ajaz Ahmad, Awais Shakoor, Bilal Ahamad Paray, Parvaiz Ahmad

**Affiliations:** 1 Plant Physiology Laboratory, Amity Institute of Biotechnology, Amity University Uttar Pradesh, Noida 201313, Uttar Pradesh, India; 2 Department of Clinical Pharmacy, College of Pharmacy, King Saud University, Riyadh 11451, Saudi Arabia; 3 Department of Environment and Soil Sciences, University of Lleida, 25198 Lleida, Spain; 4 Zoology Department, College of Sciences, King Saud University, Riyadh 11451, Saudi Arabia; bparay@ksu.edu.sa; 5 Department of Botany, Govt. Degree College, Pulwama 192301, Jammu and Kashmir, India

**Keywords:** antioxidants, *Lens culinaris*, mercury, nitric oxide, strigolactone

## Abstract

Developmental activities have escalated mercury (Hg) content in the environment and caused food security problems. The present investigation describes mercury-incited stress in *Lens culinaris* (lentil) and its mitigation by supplementation of sodium nitroprusside (SNP) and strigolactone (GR24). *Lentil* exposure to Hg decreased root and shoot length, relative water content and biochemical variables. Exogenous application of SNP and GR24 alone or in combination enhanced all of the aforementioned growth parameters. Hg treatment increased electrolyte leakage and malondialdehyde content, but this significantly decreased with combined application (Hg + SNP + GR24). SNP and GR24 boosted mineral uptake and reduced Hg accumulation, thus minimizing the adverse impacts of Hg. An increase in mineral accretion was recorded in lentil roots and shoots in the presence of SNP and GR24, which might support the growth of lentil plants under Hg stress. Hg accumulation was decreased in lentil roots and shoots by supplementation of SNP and GR24. The methylglyoxal level was reduced in lentil plants with increase in glyoxalase enzymes. Antioxidant and glyoxylase enzyme activities were increased by the presence of SNP and GR24. Therefore, synergistic application of nitric oxide and strigolactone protected lentil plants against Hg-incited oxidative pressure by boosting antioxidant defense and the glyoxalase system, which assisted in biochemical processes regulation.

## 1. Introduction

Anthropogenic activities, industrialization, urbanization, mining and application of synthetic fertilizers have accelerated the release of toxic metals into the environment [1]. Mercury (Hg) toxicity has been recognized as the most serious threat to agricultural productivity, and may become more frequent in the next few years [2,3]. The increased accretion of heavy metals in environment beyond the threshold level has shown negative impacts on soil texture, microbes and plants, and reaches animals and human beings via the food chain [4,5]. Heavy metals are stable elements that tend to accumulate in the tissues of living organisms [6]. Hg is present on the earth’s surface in very low amounts; however, industries such as cement and fluorescent lamp factories, geothermal power plants and coal mines, and their activities, such as mining and fossil fuel burning, release wastes/effluents which escalate Hg levels in the environment [7,8]. Hg is hazardous non-essential element which adversely affects the growth of plants [9,10]. Hg is commonly known as quicksilver because of its silvery tinge and swift motion. It has been considered as an environmental contaminant because of its pernicious nature, persistence and widespread pollution [11,12]. It is found in different forms, such as elemental, methyl mercury, ionic, mercury sulfide and hydroxide [13]. The hazardous nature of Hg in soil depends on its chemical speciation [11]. It has been ranked third in the category of injurious materials by the Agency for Toxic Substances and Disease Registry [14]. The safe limit of Hg ions in potable water is 10 nM to prevent ailments in human beings, as per the guidelines of the Environmental Protection Agency of the United States [15]. 

Mercuric ion has more affinity for the sulfhydryl group, and it binds to -SH groups of proteins, damages protein structure and shows expulsion of essential elements [16]. In the year 1955, residents of Minamata Bay, Japan suffered from Hg poisoning after consumption of Hg-contaminated fish and seafood; more than four hundred people died due to Minamata disease [17]. Hg toxicity induces mental retardation, abnormal behavior, muscular weakness, lesions in the colon, abdominal pain and problems in the gastrointestinal tract of affected humans [15]. Piscopo et al. [18] and Tortora et al. [19] reported that Hg induced structural and metabolic alterations in erythrocytes, with changes in the oxygen-binding capacity of hemoglobin and membrane proteins. Henriques et al. [20] stated that Hg adversely affects the reproductive and endocrine systems of both male and female human beings. 

Reactive oxygen species (ROS) such as superoxide and hydroxyl ions, hydrogen peroxide and singlet oxygen can be naturally produced in plant cells during metabolism, directly by metal stress through Haber–Weiss reactions, indirectly by NADPH oxidase stimulation or by inhibiting enzymes via essential cation displacement [21]. Excessive free radical generation occurs in plant cells under stress conditions, which disrupts the balance between ROS and antioxidant activity, damages biomolecules and disturbs the functional and structural integrity of cells [22]. The imbalance between oxidants and antioxidants leads to a disruption of redox signaling and molecular damage [22]. The plants absorb Hg from soil and water by their roots and also from the surfaces of leaves [23]. Hg is deleterious for plants, which causes redox imbalance and changes in photosynthetic activity, as well as impaired crop productivity [24]. Hg shows adverse impacts on plants even when present in very minute quantities, such as growth retardation, photosynthesis inhibition [25], imbalance of nutrients [26], free radical production, lipid peroxidation [27] and genotoxicity [28]. Plants have indigenous resistance mechanisms, such as osmolytes, antioxidants and the glyoxylase system, to mitigate metal-incited stress. The complex multilevel network of the antioxidative defense system operates to counteract harmful reactive oxygen species and maintain homeostasis within the plant cell [27]. Antioxidants represent molecules capable of quenching free radical reactions and delaying or preventing cell damage. Antioxidant enzymes not only protect various components of the cells from damage, but also play a pivotal role in plant growth and development by modulating cellular processes such as mitosis, cell elongation and differentiation, conjugation of metabolites, synthesis of proteins and nucleotides and expression of stress-responsive genes [27]. The antioxidative defense system is made up of enzymatic constituents such as superoxide dismutase (SOD), catalase (CAT), ascorbate peroxidase (APX) and glutathione reductase (GR), as well as non-enzymatic components such as reduced ascorbate and oxidized glutathione, which can neutralize ROS and protect cellular machinery [27]. Glutathione (GSH) is key component in metal scavenging due to its high affinity with thiol groups of metals; it acts as precursor for the formation of phytochelatins. Nitric oxide (sodium nitroprusside is a donor of NO), a multifunctional gaseous molecule, plays significant role in decreasing heavy metal stress by means of the antioxidant defense mechanism in plants to reduce oxidative pressure [5,29]. Asgher et al. [30] found NO function in mitigation of abiotic pressure. Utilization of nitric oxide decreased cadmium-incited oxidative pressure in *Vigna radiata* [31]. Sodium nitroprusside (SNP), an established NO donor used in plant science research as an iron-nitrosyl derivative, releases cyanide and iron in solution at high concentrations, which limits its application [32]. Strigolactones (GR24), a multifunctional butenolide molecule, functions as molecular cue with significant function in seed germination, plant development, signaling and defense to combat abiotic stresses [33,34]. Strigolactones are produced in very low amounts in plants, and they perform a dual role as both an exogenous and endogenous signaling molecule. Strigolactones promote root and root hair length and repressed lateral root development, which may help in nutrient uptake and enhanced plant growth [35]. Rehman et al. [36] reported that GR24 application promoted nodulation and increased nitrogen acquisition in legume plants such as alfalfa, pea and soybean. Strigolactone accretion has been reported in the stressed cells of plants, and this incites the generation of osmolytes and antioxidants for the maintenance of homeostasis [33]. Strigolactones work with other phytohormones and assist in regulatory networks for survival of plants under harsh conditions [37].

Lentil (*Lens culinaris* L.; family: Fabaceae) is one of the major pulses, as its annual global production is approximately 4.5 million tons. Lentil is a rich protein source and contains less lipids; because of this, its consumption reduces the risk of diabetes, heart disease and obesity, which are fatal diseases [38]. Lentil is an economically important crop, as it has a lower price as compared to another pulses and it can be used as main source of protein in developing nations. The aim of the present investigation was to analyze the response of *Lens culinaris* to Hg stress and to alleviate the impact of individual and synergistic application of SNP (a donor of nitric oxide) and GR24 on the growth, physiological process and antioxidant enzymes activities of lentil. As per our information, the combined treatment of SNP and GR24 on the antioxidant and glyoxalase systems of lentil to combat Hg stress have not yet been reported. We hypothesize that synergistic effects of nitric oxide and strigolactone can improve the morphological and biochemical attributes of lentil to increase its resistance against Hg toxicity. The results of the present investigation can be used for its further application in crop production in metal-contaminated areas.

## 2. Results

### 2.1. Growth Parameters

The exposure of Hg caused decrease in length of roots and shoots and biomass of lentil seedlings as compared to the controls. Lentil seedlings reflected 66.4, 51.5, 71.9 and 89.2% reductions in their length, fresh weight and dry weight with Hg compared to the control (Table 1). However, SNP and GR24 application induced an increase in the length of the seedlings, and SNP + GR24 combined application registered 21.6 and 10.13% increases in root and shoot length over the control lentil seedlings. As compared to Hg-exposed seedlings, Hg + SNP + GR24 treatment showed 187.6 and 607.4% enhancements in fresh and dry weight, respectively. A maximum reduction of 68.3% in relative water content was observed in Hg-treated seedlings, and RWC was increased to 89.8, 92.5 and 97% in SNP-, GR24- and SNP + GR24-treated lentil seedlings as compared to Hg-exposed seedlings. Supplementation of SNP and GR24 improved relative water content in lentil seedlings under Hg stress. Relative water content was also increased by 91.7% in lentil seedlings treated with Hg + SNP + GR24 in comparison to Hg-treated seedlings (Table 1).

### 2.2. Pigment Content

Hg stress adversely affected pigment content in lentil seedlings, whereas foliar treatment of SNP and GR24 showed a significant increase in chlorophyll content. The co-application of SNP and GR24 significantly enhanced total chlorophyll content by 5 and 10.6%, and carotenoids by 8.2 and 14.3%, respectively, over the control (Table 2). The total chlorophyll and carotenoid contents were significantly increased by 14.8 and 18.4%, respectively, in the combined treatment with SNP + GR24 in comparison to the control. A maximum carotenoid content of 34.9% was reported in lentil seedlings with SNP + GR24 treatment for Hg stress. Thus, SNP and GR24 application decreased the decline in pigment content, with maximum amelioration observed with the combined treatment (SNP + GR24), in lentil seedlings.

### 2.3. Osmolytes Content

Sodium nitroprusside (NO donor) and strigolactone (GR24) application individually and synergistically improved accumulation of sugar, proline, glycine betaine and protein in lentil seedlings. The increase in sugar content in lentil seedlings in Hg + SNP + GR24 treatment may be due to the increase in photosynthesis. Hg-treated seedlings showed increases of 26.7 and 164.81% in proline and glycine betaine content compared to the control. Proline and glycine betaine act as osmolytes. Proline accumulates in plant cytosols when they are exposed to metals, and also participates in osmotic adjustment, biomolecule stabilization, chelation of metals and removal of free radicals, whereas glycine betaine improves plant growth by combating metabolic dysfunction during stress. Increases in the accumulation of these osmolytes protect lentil seedlings under Hg stress. Significant stimulation in osmolyte content, namely 72.5, 215.5, 234.8 and 8.83% in sugar, proline, glycine betaine and protein, respectively, were reported in lentil seedlings with Hg + SNP + GR24 treatment as compared to the control (Table 3). Protein content was significantly affected by Hg exposure, as a maximum reduction of 38.9% in protein content was observed in lentil seedlings over the control, but protein content was enhanced to 46.3 and 59.6% with SNP and GR24 treatment, respectively, under Hg stress. 

### 2.4. Oxidative Stress Markers

**Figure 1:**
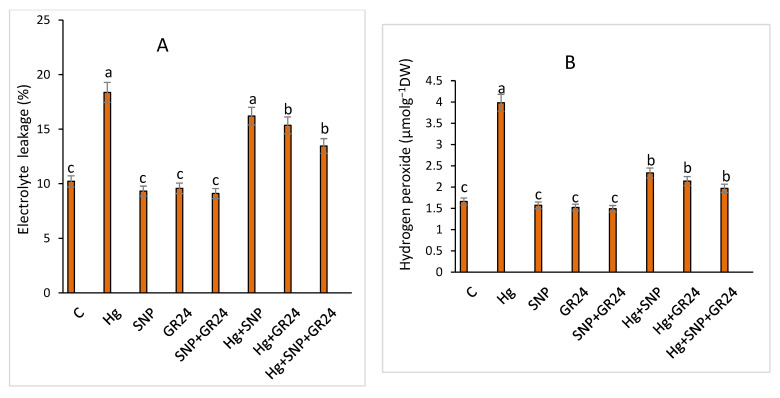

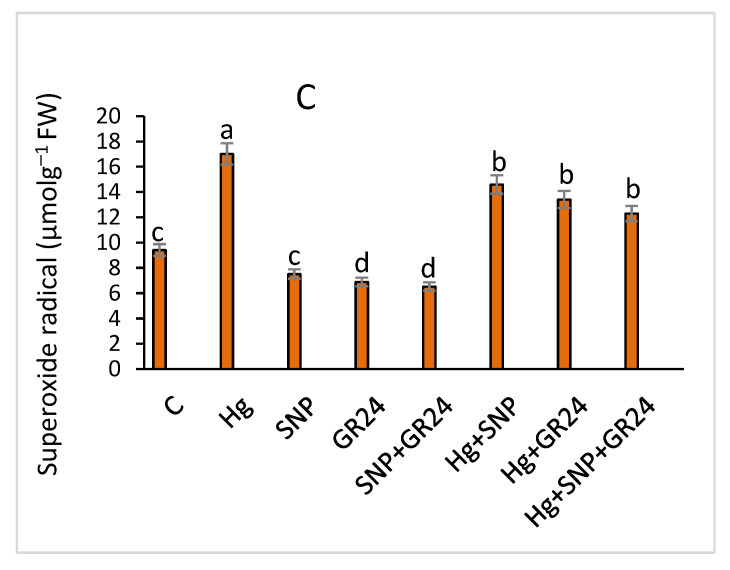
Electrolyte leakage (**A**), hydrogen peroxide (**B**) and superoxide contents (**C**) in *Lens culinaris* L. grown under Hg stress with and without SNP and GR24. Various letters on bars show variation among treatments at a *p* < 0.05 significance level according to ANOVA and DMRT. SNP = 100 µM and GR24 = 100 µM concentrations.

Hg stress enhanced electrolyte leakage by 79.7, production of H_2_O_2_ by 139.8 and superoxides by 80.8% in *Lens culinaris* L. in comparison to the control. The supplementation of SNP and GR24 reduced electrolyte leakage by 49.3 and 47.8%, respectively, in comparison to Hg treatment. Synergistic application of SNP + GR24 significantly reduced electrolyte leakage, hydrogen peroxide and superoxide production, and thus decreased oxidative pressure in lentil seedlings (Figure 1). 

**Figure 2:**
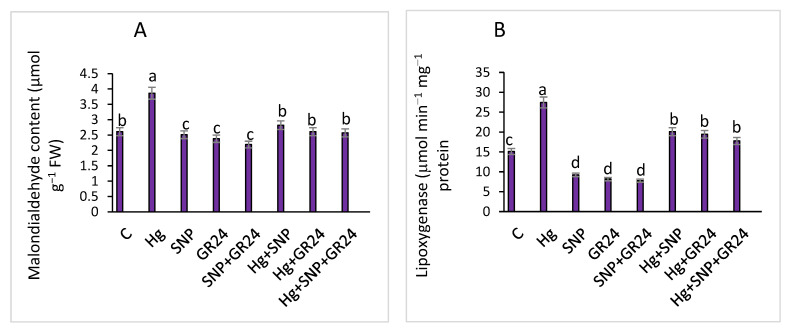
Lipid peroxidation (**A**) and lipoxygenase (**B**) activityin *Lens culinaris* L. grown under Hg stress, with and without SNP and GR24. Values above the bars with different letters show variation among treatments at a *p* < 0.05 significance level according to ANOVA and DMRT. NO = 100 µM and GR24 = 100 µM concentrations.

MDA is marker of oxidative stress, and a higher MDA content was observed under Hg stress. However, its content was reduced with SNP and GR24 treatment and lipoxygenase activity in lentil seedlings compared to the control. Lentil seedlings treated with SNP and GR24 exhibited a decrease in lipoxygenase activity, with a maximum decline of 71.6% with SNP + GR24 treatment and Hg treatment (Figure 2). 

### 2.5. Accumulation of Hg and Other Inorganic Elements

Hg accumulated both in roots and shoots after treatment. In our investigation, a 60-fold rise in the amount of Hg was reported in lentil roots, in contrast to a 56-fold enhancement in shoots, as compared to the control. A significant reduction in translocation factor value 0.75 occurred in combined treatment with SNP + GR24, and reflected a reduction in Hg translocation from roots to shoots (Table 4). Treatment of SNP and GR24 reduced Hg accumulation in roots by 45.4 and 48.6%, respectively, over Hg stress. A greater uptake of minerals such as nitrogen, phosphorus, potassium, magnesium and calcium was recorded by lentil roots in comparison to shoots (Table 5). The following trend was observed in uptake of minerals by lentil roots and shoots: K > N > P > Mg > Ca. The results revealed that in the presence of Hg, the contents of N, K, P, Mg and Ca declined in lentil roots by 36.6, 68.5, 46.8, 69.5 and 68.6%, respectively, compared to the control seedlings. Application of SNP and GR24 enhanced the growth parameters of plants, which may possibly be due to the improvement in uptake of nutrients.

### 2.6. Methylglyoxal Content and Glyoxalase Enzyme Activity

**Figure 3:**
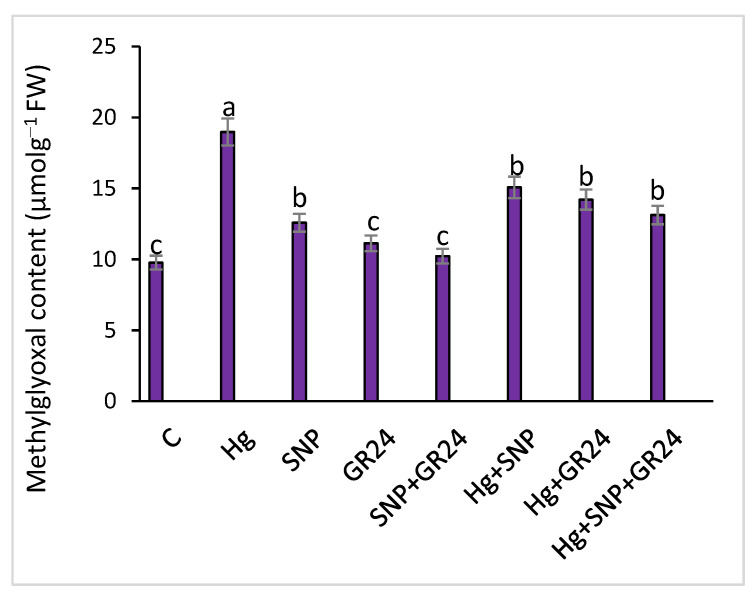
Methylglyoxal content in *Lens culinaris* L. grown under Hg stress, with and without SNP and GR24. Values above the bars with different small letters show significant differences among treatments at a *p* < 0.05 significance level, according to ANOVA and DMRT. SNP = 100 µM and GR24 = 100 µM concentrations.

Hg stress induced high methylglyoxal production (94%) in comparison to the control. The exogenous supply of SNP and GR24 reduced methylglyoxal content, with maximum reduction of 46%, in SNP + GR24 treatment under Hg stress, individually as well as synergistically (Figure 3). Methylglyoxal, an important biomarker for plant stress responses, was produced two to six times more under metallic stress in comparison to non-stress conditions [39]. 

**Figure 4:**
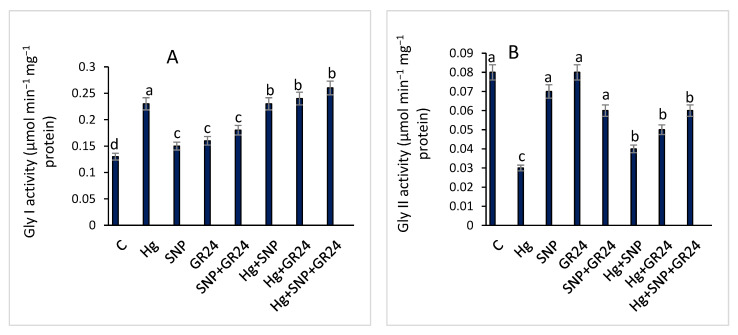
Glyoxalase I (**A**) and Glyoxalase II (**B**) activities in *Lens culinaris* L. grown under Hg stress, with and without SNP and GR24. Values above the bars with different small letters show significant differences among treatments at a *p* < 0.05 significance level, according to ANOVA and DMRT. SNP = 100 µM and GR24 = 100 µM concentrations.

Hg treatment increased glyoxylase I activity by 76.9%, but decreased glyoxylase II by 62.5%, in lentil plants compared to the control. Supplementation of SNP and GR24 in lentil seedlings containing Hg significantly enhanced Gly I activity by a maximum of 13% in Hg + SNP + GR24 treatment as compared to Hg alone. The exogenous SNP and GR24 supplementation along with Hg stimulated 100% of the Gly II activities in Hg-treated lentil seedlings (Figure 4).

### 2.7. Antioxidant Enzymes

**Figure 5:**
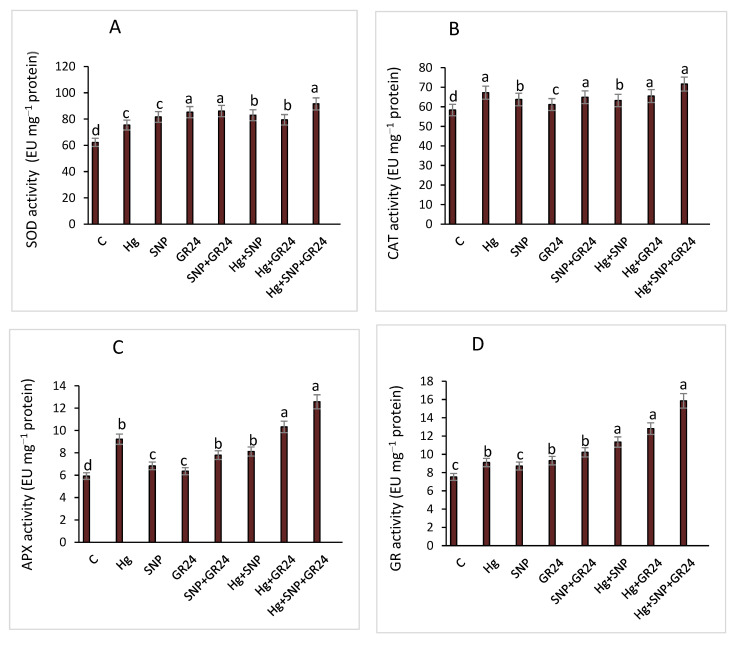
Superoxide dismutase (**A**), catalase (**B**), ascorbate peroxidase (**C**), and glutathione reductase (**D**) enzymes in *Lens culinaris* L. grown under Hg stress, with and without SNP and GR24. Values above the bars with different small letters show significant differences among treatments at a *p* < 0.05 significance level, according to ANOVA and DMRT. SNP = 100 µM and GR24 = 100 µM concentrations.

After exposure to SNP and GR24, either individually or in combination, antioxidant enzymes such as SOD, CAT, APX and GR reflected different trends in the lentil seedlings. Hg stress enhanced antioxidant enzymes activities significantly compared to the control, and a further increase was observed when SNP and GR24 were supplemented to Hg-treated seedlings. The synergistic application of SNP + GR24 enhanced SOD and GR by 14 and 12.3%, respectively, in lentil seedlings under Hg treatment. However, 3.5 and 15.5% reductions in CAT and APX content were reported in lentil seedlings. Lentil seedlings treated with Hg + SNP + GR24 showed increases in SOD, CAT, APX and GR of 21.4, 6.6, 36.3 and 74.2%, respectively, as compared to Hg y-treated lentil plants (Figure 5). The synergistic impact of SNP and GR24 was more pronounced in comparison to the individual treatment. 

**Figure 6:**
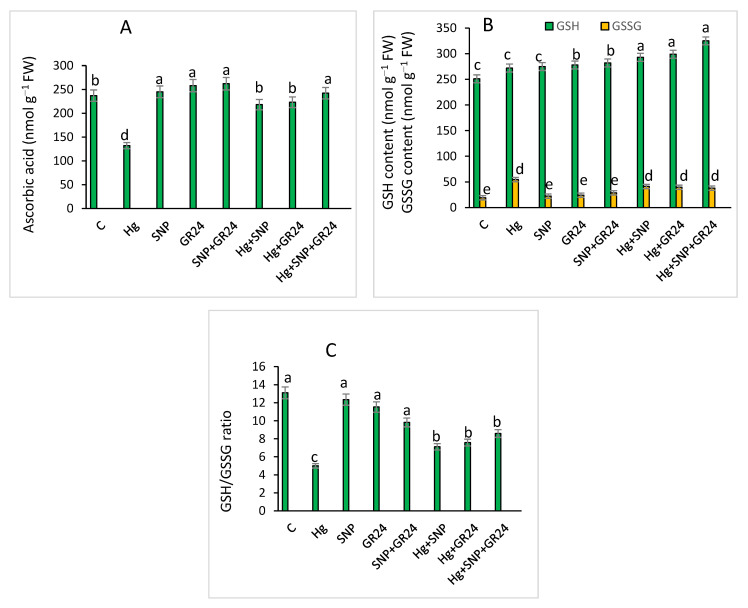
Contents of ascorbate (**A**), reduced glutathione and oxidized glutathione (**B**) and ratio of GSH/GSSG (**C**) in *Lens culinaris* L. grown under Hg stress with and without SNP and GR24. Values above the bars with different small letters represent significant differences among treatments at a *p* < 0.05 significance level, according to ANOVA and DMRT. SNP = 100 µM and GR24 = 100 µM concentrations.

Hg stress reduced the accumulation of ascorbate, but increased glutathione content. However, SNP and GR24 enhanced the amounts of ASA and GSH in Hg-treated seedlings. Synergistic treatment with SNP and GR24 enhanced ascorbate by 10.5% and glutathione by 11% in lentil seedlings compared to the control (Figure 6). The oxidized glutathione content (GSSG) was enhanced by 184.2% with Hg treatment as compared to the control. The significant inhibition of 62% in the GSH/GSSG ratio was observed with Hg treatment, over the control. However, SNP and GR24 supplementation increased the GSH/GSSG ratio, with 72% stimulation with Hg + SNP + GR24 treatment compared to Hg-stressed seedlings (Figure 6).

## 3. Discussion

The exposure of Hg significantly reduced the growth and biochemical parameters of lentil plants. In this investigation, Hg showed inhibition in length and biomass of lentil seedlings as compared to the control. Seedling growth is a significant marker for analyzing plants’ responses under stress conditions. Hg + SNP + GR24 treatment induced 187.6 and 607.4% enhancements in fresh and dry weight, respectively, in comparison to Hg-treated lentil seedlings. Iqbal et al. [40] reported phytotoxic effects of Hg on seed germination and seedling growth of *Albizia lebbeck*. Accumulation of ROS depletes ATP and adversely affects respiration rate and plant growth [41]. The Hg-mediated inhibition of growth was due to the interaction of Hg with sulfhydryl groups of proteins, which form S-Hg-S bridges and show alterations in enzymatic activities and protein structures [42]. Hg induced a decline in mitotic activity, reduced elongation in cells and caused loss of cell turgidity. Due to the complex dissociation constant of ATP-Hg as compared to ATP-Mg, it also decreased the growth of plants under metal stress [3]. Mei et al. [13] reported that Hg is able to bind with proteins of water channels in root cells, creating a barrier to the flow of water and thus affecting transpiration and inhibited uptake and transport of nutrients in cotton seedlings. An Hg-induced loss of function of aquaporin was recorded by Magistrali et al. [43]. A maximum reduction of 68.3% in RWC was observed in Hg treated seedlings; however, it escalated to 89.8, 92.5 and 97% with SNP, GR24 and SNP + GR24 treatments, respectively. The decline in relative water content in the presence of Hg was due to a reduction in hydraulic conductivity and cell turgor [44]. Nitric oxide and strigolactone induced an enhancement to the water content and improved the extensibility of the cell wall, with an increase in cell division and physiological variables, in lentil seedlings (Table 1).

In our study, Hg was accumulated more significantly in roots, and less was translocated towards shoots [13]. Higher accumulation of Hg was recorded in lentil roots, because the roots are the primary organs to have contact with Hg. However, a significant reduction in translocation factor value 0.75 was observed with SNP + GR24 treatment, which showed inhibition in the transfer of Hg from roots to shoots and protected lentil seedlings from Hg stress (Table 4). Hg indirectly affected plant growth by interrupting nutrient absorption. There are very few studies available on the relationship between Hg and uptake of nutrients by plants. It has been reported that Hg may affect nitrogen assimilatory enzymes and nitrate uptake, and may decrease the concentration of nitrogen in plants. Potassium is a stress-resistant nutrient that assists plants with adapting under adverse conditions. Magnesium has a significant function in the photosynthesis process, as it is an important component for chlorophyll synthesis. Supplementation of SNP- and GR24-enhanced lentil plant growth may be due to escalation in mineral uptake (Table 5). The same results were recorded by Ahmad et al. [27]. Nitric oxide improved N, P and K uptake in wheat under cadmium treatment [45]. Nitric oxide promoted Ca^2+^ influx, increased calcium transporters and maintained the level of calcium in cells [46]. Uptake of Ca and K was enhanced with a reduction in Na/K ratio by the application of nitric oxide [47].

Nitric oxide is generated from sodium nitroprusside, which promotes defensive responses and regulates biochemical processes in plants under harsh conditions [48,49]. Nitric oxide functions as an endogenous signaling molecule and assists in the coordination of the signaling network [50]. The utilization of NO decreased heavy metal aggregation in plant cells, such as chromium in maize [51], nickel in rice [52] and cadmium in barley [53]. Strigolactones play a significant role in the regulation of biochemical processes for plants’ survival under abiotic stresses [34]. Kolbert [54] observed that exogenous strigolactone application increased nitric oxide content. Similar findings have been reported in our experiments (Table 1). Strigolactones acted as rhizosphere signals and promoted plant and arbuscular mycorrhizal fungi symbioses by increasing the germination of spores and hyphal branching [55]. They may provide moisture and nutrients to plants under nutrient deprivation.

The pigment content and rate of photosynthesis were decreased in plants due to their exposure to high Hg levels [56]. A significant reduction of 41% was recorded in the total chlorophyll content of lentil seedlings under Hg stress as compared to the control. However, combined treatment of SNP and GR24 enhanced the total chlorophyll content in lentil seedlings under Hg treatment. The decrease in the chlorophyll amount by HgCl_2_ was due to a decrease in Mg^2+^ and Fe^2+^ absorption or metal ion displacement by Hg [16]. Hg can reduce chlorophyll content by enhancing chlorophyllase enzyme activity, oxidation/reduction of chlorophyll molecules by metal-induced free radicals and replacement of Mg^2+^ in the porphyrin ring of chlorophyll molecules by Hg [57]. It has been predicted that Hg binds to cystein residues with high affinity. Therefore, all enzymes that contain cystein residue in their catalytic centers are affected. The thioredoxin-dependent redox control of enzyme activity is also affected, thus affecting most of the light-controlled catalytic events. High concentrations of mercuric chloride adversely affected growth, chlorophyll and antioxidant enzymes of *Lemna gibba*, *Lemna minor* and *Spirodela polyrhiza* [58]. Min et al. [59] found decreased chlorophyll in *Vitis vinifera* under drought stress, whereas GR24 supplementation maintained chlorophyll content.

MDA is a marker of lipid peroxidation and its content reflects oxidative pressure in plants [60]. Hg exposure incited oxidative strain, which was reflected by increased MDA and H_2_O_2_ levels. However, SNP and GR24 application reduced H_2_O_2_ and MDA levels. In this study, the results revealed that escalated H_2_O_2_ generation was consistent with electrolyte leakage and lipid peroxidation in Hg-treated lentil seedlings [61]. The significant enhancement in H_2_O_2_ levels was observed in lentil seedlings under Hg stress, but combined treatment (SNP + GR24) significantly reduced H_2_O_2_ levels. Kaya and Ashraf [62] observed NO function in tomato plants against boron-incited oxidative pressure by reducing free radicals.

The AsA-GSH cycle inhibits the accumulation of hydrogen peroxide in the cells, and ascorbate peroxidase reduces it to water [63,64]. Combined treatment with SNP + GR24 maintained water potential by enhancing proline and sugar contents by 79.6 and 120% under Hg treatment. Hg treatment increased proline content, but a marked increase was observed with SNP and GR24 co-supplementation to lentil seedlings (Table 3). Reddy et al. [65] stated that proline protects photosynthetic machinery and preserves cellular integrity under oxidative damage. In the present study, utilization of SNP and GR24 showed enhancement in non-enzymatic antioxidants, which were reduced in the presence of mercury. To avoid the deleterious impacts of mercury, osmolytes accumulated for maintenance of cellular function by increasing water content in cells, which assists in scavenging ROS and maintains enzyme function [66]. 

Less synthesis of proteins in presence of Hg might be due to a deficiency in the sugars or nutrients essential for protein synthesis, such as magnesium and potassium, or to increased protease activity or damage to the photosynthetic system [58]. In the present investigation, protein content was significantly reduced by Hg exposure, as a maximum reduction of 38.9% in protein content was observed in lentil seedlings compared to the control (Table 3). Hg rapidly attacks different biomolecules and interrupts cell metabolism [24]. The osmolytes, glyoxylase and antioxidant defense system are present in plant cells, which ensures the detoxification of free radicals, reduces membrane lipid peroxidation and prevents the breakdown of protein and nucleic acids by delaying oxidation under stress [67]. Methylglyoxal is formed via the glycolytic pathway in plant cells, but its excessive generation retards the growth of cells and promotes the deterioration of protein and lipids [68]. Suhartono et al. [69] observed two to six times greater production of methylglyoxal under metal toxicity. Apart from antioxidants, the glyoxalase system plays a great role in the detoxification of methylglyoxal via glyoxalase I and II enzymes [68]. Our study reflected that Hg treatment enhanced the activity of glyoxalase I and reduced that of glyoxalase II (Figure 4). The combined application of SNP and GR24 regulated the function of both glyoxalase enzymes and showed that methylglyoxal was efficiently detoxified in lentil seedlings (Figure 4).

Accretion of enzymatic and non-enzymatic antioxidants has been reported in *Brassica juncea* under cadmium stress [70]. Nitric oxide acts as an antioxidant, and NO-treated plants eliminate free radicals by up-regulation of the antioxidant system and enhance cell membrane stability [48]. Our results demonstrated that SNP and GR24 mediated a rise of 21.4, 6.6, 36.3 and 74.2% in SOD, CAT, APX and GR activities, respectively, which was associated with stress resistance and decreased intensity of the oxidative stress generated by Hg [27]. Ascorbate is a water-soluble antioxidant, similarly toas reduced glutathione, and these check for cell damage caused by free radical aggregation. Xu et al. [71] found that NO induced ascorbate and reduced glutathione accumulation under cadmium stress in groundnut plants. APX plays a pivotal role in the ascorbate–glutathione pathway for plant defense by scavenging hydroxyl radicals with ascorbate expenditure. SOD removes superoxide ions, while catalase converts hydrogen peroxide into water and oxygen [72]. The AsA-GSH cycle regulates hydrogen peroxide levels. Pirzadah et al. [73] observed that APX and GR activities were enhanced with Hg treatment, whereas SOD and CAT were reduced in *Fagopyrum tataricum* with a high concentration of Hg. Figure 6C revealed that nitric oxide and strigolactone up-regulated GSH/GSSH ratio [47]. The balance between GSH and GSSG is an important factor in maintaining the redox condition of the cell, as under metal stress, reduced glutathione is converted into an oxidized form. Ascorbate and reduced glutathione act as redox buffering agents, maintain cellular redox state and preserve plasma membrane integrity. Hg-treated lentil seedlings with SNP and GR24 application enhanced reduced glutathione content (Figure 6B). Glutathione reductase converts GSSG to GSH and regulates the GSH/GSSG ratio, which is required for cellular homeostasis.

**Figure 7:**
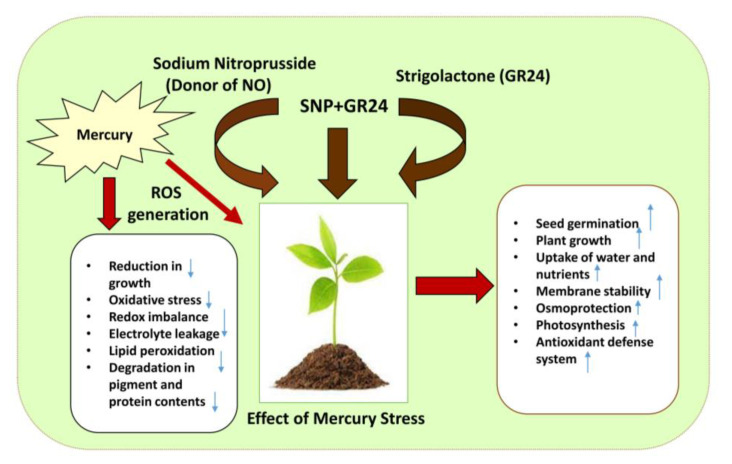
Schematic presentation of effect of Hg stress on lentil plants and the alleviating role of SNP and GR24 for plant protection. Arrow (↑) indicates increase whereas (↓) indicates reduction in physiological processes.

Hg treatment showed excessive generation of free radicals and disrupted protein molecules in *Melissa officinalis* [16]. Supplementation of SNP reduced oxidative pressure and lipid peroxidation by reduction in lipoxygenase activity and increased activity of antioxidant enzymes [74]. Nitric oxide maintained pigment synthesis, redox homeostasis and antioxidant defense system enzymes by inhibiting copper toxicity in barley [74]. Nitric oxide and strigolactone can mitigate Hg toxicity by maintaining a glutathione–ascorbate cycle and an antioxidant defense system which protects proteins by modulating redox homeostasis in cells [74]. The protective role of SNP and GR24 against an Hg-inspired oxidative strain might be because of the modulation of antioxidant and glyoxalase enzyme activities. The increase in GR activity under SNP and GR24 treatment converted ascorbate and glutathione into their reduced form, which inhibited Hg-induced oxidative damage and ensured normal growth. Hence, synchronized regulation of glyoxalase and antioxidant pathway enzymes was recorded for notable resistance in lentil plants to counter oxidative stress generated by Hg. Figure 7 displays a summary of the negative effects triggered by Hg stress in lentil seedlings and how the exogenous application of NO and GR24 can reverse/palliate these damages.

## 4. Materials and Methods

### 4.1. Procurement of Chemicals

Mercuric chloride (HgCl_2_; molecular weight: 271.52 g·mol^−1^), sodium nitroprusside (SNP; C_5_FeN_6_Na_2_O; molecular weight: 261.918 g·mol^−1^) and synthetic strigolactone analog (GR24; C_17_H_14_O_5_; molecular weight: 298.29 g·mol^−1^) were purchased from Merck, India. Analytical grade reagents were used for the experiments.

### 4.2. Cultivation of Plants and Stress Treatment

Lentil seeds (*Lens culinaris* L. variety NDL-2) were purchased from the seed agency of Noida, India. Lentil seeds were treated with 10% sodium hypochlorite solution for five minutes, then cleaned with deionized water. Lentil seeds were sown in nutrient-rich substrate (N:P:K 4:2:1) in a tray for 10 days and irrigated properly, and then 2 groups of pots were prepared and supplemented with mercuric chloride (HgCl_2_) (0 mg for control and 150 mg/kg soil) for Hg treatment. In treatment, each plastic pot (14 cm height × 8 cm diameter) contained 150 gm soil and around 22.5 mg mercuric chloride. A single lentil seedling (10 days old) was transferred to each plastic pot, and these were kept under 150 μmol photons m^−2^·s^−1^ photosynthetically active radiation for a 16-hour day and 8-hour night regime at 23 ± 2 °C under 90% relative humidity. Strigolactone (GR24) solution was prepared in ethanol. After 4 days of seedling establishment, a solution of 100 μM SNP (donor of NO) and 100 μM GR24 (10 mL per pot) was sprayed individually onto the lentil seedlings with a hand sprayer (manually operated with pressure 25 psi, kept 20 cm above the plants) 5 times in a week until 15 days had passed. Approximately 100 mL of SNP and GR24 solution, respectively, were given to lentil seedlings as per their treatment, and untreated seedlings were sprayed with distilled water. After repeated experiments with a wide range of concentrations of SNP and GR24, concentrations and application methods were optimized for SNP and GR24 for further experiments [75]. The experimental scheme contained the following seven combinations: (1) Control; (2) Hg; (3) 100 µM SNP; (4) 100 µM GR24; (5) Hg +100 µM SNP; (6) Hg +100 µM GR24; (7) Hg +100 µM SNP +100 µM GR24. Hence, at the vegetative stage, twenty-five-day-old lentil seedlings (after SNP and GR24 treatment for two weeks) were harvested and analyzed for biochemical variables, as given below:

### 4.3. Growth and Relative Water Content

The length and biomass of lentil seedlings were measured in both the control and treated plants by the method described by Alsahli et al. [76]. For RWC, lentil leaves were cut into uniform sizes, fresh weight was taken and a disc was placed in distilled water at 25 ± 2 °C for 12 h under dark conditions. After measurement of the turgid weight, dry weight was analyzed by placing the discs into the oven at 80 °C for 48 h.

RWC = (FW − DW)/(TW − DW)(1)

### 4.4. Pigment Content

Leaves from the control and treatment plants were grounded with 80% acetone and centrifuged. The supernatant’s optical density was measured with a spectrophotometer at 663, 646 and 470 nm for estimation of total chlorophyll and chlorophylla, b and carotenoids, as per the method of Lichtenthaler [77].

Total chlorophyll (mg/g) = (20.2 × OD_645_ + 8.02 × OD_663_) × V/100 × W

Chlorophyll a (mg/g) = (12.7 × OD_663_ − 2.69 × OD_645_) × V/100 × W

Chlorophyll b (mg/g) = (22.9 × OD_645_ − 4.68 × OD_663_) × V/100 × W

Carotenoid (mg/g) = (1000 OD_470_ − 1.82 Chl_a_ − 85.02 Chl_b_)/198

where V = volume of the supernatant in ml, W = fresh weight of the leaves in g Chl_a_ = chlorophyll a, Chl_b_ = chlorophyll b and OD = optical density.

### 4.5. Evaluation of Sugar, Proline, Glycine Betaine and Protein Contents

Total soluble sugar was analyzed with the Hedge and Hofreiter [78] procedure. Lentil leaves were crushed in ethyl alcohol, and an anthrone reagent was added into the supernatant and kept boiling for ten minutes. The absorbance was taken at 620 nm, and the amount of sugar was assessed by standard curve of glucose.

Proline content was analyzed with the procedure of Bates et al. [79]. Sulphosalicylic acid (3%) was used for the crushing of leaves; filtrate was incorporated with acetic acid and acid ninhydrin and kept at 100 °C for one hour. After the addition of toluene into the mixture, absorbance was taken at 520 nm.

Glycine betaine amount was analyzed by Grieve and Grattan [80]. After the reaction with the potassium iodide–iodine reagent at a low pH, periodide crystals were determined at 365 nm and the amount was calculated by the standard curve of glycine betaine.

Lentil leaves were crushed with 1 mL of 1N NaOH and kept at 100 °C for five minutes. The alkaline copper reagent was mixed and kept at room temperature for ten minutes; after that, a Folin–Ciocalteau reagent was added. After half an hour, at 650 nm, absorbance was measured and the protein amount was assessed with a BSA standard curve [81].

### 4.6. Measurement of Electrolyte Leakage, Hydrogen Peroxide, Superoxide Radical 

The method of Dionisio-Sese and Tobita [82] was applied for electrolyte leakage assessment. Lentil leaf discs 5 millimeters in size (100 mg) were kept in a water bath in test tubes containing deionized water (10 mL), maintained at 32 °C. After two hours, initial electrical conductivity of the medium (EC_1_) was measured using an electrical conductivity meter. Then samples were autoclaved at 121 °C for 20 min to completely kill the tissues and release all electrolytes. Samples were then cooled to 25 °C and final electrical conductivity (EC_2_) was measured. 

The electrolyte leakage was calculated by following the formula:

EL = EC_1_/EC_2_ × 100 (2)

Hydrogen peroxide content was measured by Velikova et al. [83]. At 390 nm, absorbance was taken and a standard curve was used for estimation of the hydrogen peroxide concentration. This was denoted as nmol g^−1^ FW.

For the measurement of superoxide concentration, lentil leaves were crushed with 65 mM potassium phosphate buffer and centrifugation was performed at 5000× *g*. The hydroxylamine hydrochloride (10 mM) was mixed in the supernatant, and after twenty minutes, sulfanilamide and naphthylamine were added. At 530 nm, optical density was measured and a sodium nitrite standard curve was used to calculate the superoxide content [84].

### 4.7. Lipid Peroxidation and Lipoxygenase Activity

Lipid peroxidation analysis was conducted in lentil leaves by the method of Zhou and Leul [85]. Lipid peroxidation was measured as the malondialdehyde (MDA) content based on the production of thiobarbituric acid reactive substances (TBARS). Leaves (0.5 g) were homogenized with 0.5% TBA (10 mL) and the homogenate was kept at 95 °C for half an hour and centrifuged at 5000 rpm for 8 min after cooling. The optical density of the filtrate was measured at 532 and 600 nm. 

The content of malondialdehyde was calculated by the given formula:

MDA (n mol/g FW) = (OD_532_ − OD_600_) × A × V/a × E × W (3)

where A = reaction solution volume, V = phosphate buffer solution volume, a = enzyme extract volume, W = leaves fresh weight and E = MDA constant (1.55 × 10^−1^).

Lipoxygenase activity was measured by the method of Doderer et al. [86]. The substrate was linoleic acid. At 234 nm, absorbance was measured, and a 25 mM^−1^ cm^−1^ extinction coefficient was applied for analysis.

### 4.8. Assessment of Hg and Other Inorganic Elements

Dry roots and shoots of lentil seedlings (500 mg) were treated with H_2_SO_4_ and HNO_3_ (1:5, *v*/*v*) at 60 °C for one day. The digested substance was analyzed again with a mixture of HNO_3_ and HClO_4_ (5:1, *v*/*v*). After that, the contents of Hg and minerals in the roots and shoots were analyzed by an atomic absorption spectrophotometer and expressed as μg·g^−1^ DW. 

The concentration of Hg in tissues and its translocation can be calculated by the following formulae:

Concentration Index = Hg content in treatment/Hg content in control(4)

Translocation Factor = Hg content in shoot/Hg content in root(5)

### 4.9. Antioxidant Enzymes

Enzyme extracts were prepared by crushing lentil leaves with 0.1 M sodium phosphate buffer and polyvinylpyrrolidone. After centrifugation (14,000× *g*) at 4 °C for half an hour, the supernatant was taken for analyses.

### 4.10. Superoxide Dismutase Assay

Superoxide dismutase was evaluated with the NBT photochemical procedure [87]. The mixture was composed of EDTA (0.15 mM), methionine (20 mM), riboflavin (13 µM), NBT (0.12 mM), sodium carbonate (0.05 M) and enzyme (0.4 mL). Test tubes were kept under light for half an hour, and a similar set of unilluminated assay mixtures was treated as blank. At 560 nm, nitroblue tetrazolium photoreduction was determined and analyzed with a blank sample. Superoxide dismutase (1 unit) activity can be considered an enzyme needed for 50% reduction in NBT.

### 4.11. Catalase Assay

Catalase activity was assessed by H_2_O_2_ degradation for 1 min at 240 nm by applying the extinction coefficient 39.4 mM^−1^ cm^−1^ [88]. 

### 4.12. Ascorbate Peroxidase Assay

APX estimation was performed by the method of Nakano and Asada [89]. The mixture contained 0.1 mM EDTA, 0.25 mM ascorbate, 25 mM potassium phosphate buffer, 1 mM hydrogen peroxide and 0.2 mL enzyme extract. The optical density was taken at 290 nm for 1 min with a 2.8 mM^−1^ cm^−1^ extinction coefficient. 

### 4.13. Glutathione Reductase 

Glutathione reductase was assessed by analyzing the variation in optical density at 340 nm, up to 3 min. The mixture was formed with 1 mM EDTA, 50 µM NADPH, 100 mM potassium phosphate buffer, 100 µM oxidized glutathione and 100 µL enzyme. Activity was denoted by U mg^−1^ protein by the procedure of Foster and Hess [90].

### 4.14. Assay of Glyoxalase I and II

Glyoxalase I activity was analyzed by the method described by Hasanuzzaman [91]. The mixture was made up of K_2_HPO_4_ buffer (100 mM), reduced glutathione (1.7 mM), methylglyoxal (3.5 mM) and magnesium sulphate (15 mM), and optical density was determined at 240 nm for 1 min by 3.37 mM^−1^ cm^−1^ extinction coefficient. The procedure described by Kaya et al. [92] was used to measure glyoxalase II activity. The solution contained 100 mM Tris-hydrochloride buffer, 0.2 mM DTNB and 1 mM S-D-lactoylglutathione. Activity was measured by applying a 13.6 mM^−1^ cm^−1^ extinction coefficient.

### 4.15. Methylglyoxal Content

Lentil leaves were fused with 5% HClO_4_, and after centrifugation at 11,000× *g,* the supernatant was treated with charcoal and nullified by adding potassium carbonate, N-acetyl-L-cysteine and sodium dihydrogen phosphate. Synthesis of N-α-acetyl-S-(1-hydroxy-2-oxo-prop-1-yl) cysteine was noted at 288 nm by the method of Wild et al. [93].

### 4.16. Determination of Ascorbate and Reduced Glutathione

The ascorbate amount was measured by mashing lentil leaves in TCA (6%), and 2% dinitrophenylhydrazine and 10% thiourea were mixed in the supernatant. After heating for 15 min, proper cooling was carried out at room temperature; then, 80% sulfuric acid was incorporated. Absorbance was measured at 530 nm and the analysis was conducted by the standard curve of ascorbate [94].

The reduced glutathione content was analyzed by the Ellman [95] procedure. Lentil leaves were crushed in phosphate buffer and 5,5-dithiobis-2-nitrobenzoic acid was mixed in filtrate. Absorbance was measured at 412 nm and reduced glutathione content was estimated with the reduced glutathione standard curve. 

### 4.17. Statistical Analysis

Treatments were arranged with three replicates in a randomized block design. Data were determined using ANOVA and SPSS software. The treatment mean was assessed by DMRT at *p* < 0.05.

## 5. Conclusions

The results of this study clearly indicate the potential function of exogenously applied SNP and GR24 in alleviation of adverse effects of Hg on lentil plants. SNP and GR24 have emerged as promising signaling molecules which have the ability to mitigate the deleterious impacts of Hg stress. Hg accumulation was decreased in lentil roots and shoots, and increased activities of antioxidant and glyoxylase enzymes by SNP and GR24 supplementation were responsible for the protection of lentil plants against Hg stress. The processes regulated by the stimulatory effects of SNP and GR24, related to their multi-faceted nature, could work separately or incorporated into one strategy to promote the growth of lentil plants in response to Hg stress. Nitric oxide and strigolactones have potential agronomical applications and, hence, can be used for crop growth in metal-contaminated areas. However, it is recommended that toxicological tests be conducted. The detailed molecular mechanism of crosstalk between SNP and GR24, as well as the fine-tuning of the complex signaling networks in response to Hg stress tolerance in lentil plants, needs to be further investigated in the future. Therefore, in-depth research is required on genetic engineering approaches to modulate the endogenous levels of SNP and GR24, which can serve to mitigate stresses in plants.

## Figures and Tables

**Table 1:** Impact of Hg on length of seedlings, biomass and relative water content of *Lens culinaris* L. variety NDL-2, with or without SNP and GR24.

Treatment	Root Length (cm)	Shoot Length (cm)	Fresh Weight (g)	Dry Weight (g)	Relative Water Content (%)
Control	12.76 ± 0.36 ^a^	26.15 ± 0.17 ^a^	8.95 ± 0.05 ^b^	2.49 ± 0.23 ^a^	97.58 ± 0.15 ^a^
Hg	4.29 ± 0.24 ^c^	12.69 ± 0.36 ^c^	2.51 ± 0.06 ^d^	0.27 ± 0.05 ^c^	68.29 ± 0.35 ^c^
SNP	13.81 ± 0.23 ^a^	26.46 ± 0.39 ^a^	9.34 ± 0.34 ^a^	2.55 ± 0.19 ^a^	89.81 ± 0.06 ^b^
GR24	14.92 ± 0.09 ^a^	27.70 ± 0.35 ^a^	10.02 ± 0.03 ^a^	2.67 ± 0.18 ^a^	92.45 ± 0.04 ^a^
SNP + GR24	15.51 ± 0.35 ^a^	28.80 ± 0.24 ^a^	10.54 ± 0.32 ^a^	2.8 ± 0.07 ^a^	96.99 ± 0.35 ^a^
Hg + SNP	8.69 ± 0.13 ^b^	15.37 ± 0.45 ^b^	5.76 ± 0.29 ^c^	1.35 ± 0.09 ^b^	83.84 ± 0.38 ^b^
Hg + GR24	9.58 ± 0.37 ^b^	16.73 ± 0.33 ^b^	6.61 ± 0.09 ^b^	1.54 ± 0.03 ^b^	88.32 ± 0.07 ^b^
Hg + SNP + GR24	11.63 ± 0.22 ^b^	19.87 ± 0.15 ^b^	7.22 ± 0.19 ^b^	1.91 ± 0.07 ^b^	91.71 ± 0.08 ^a^

Data presented are mean ± sem (*n* = 3). Different letters on values show significant variation among treatments at a *p* < 0.05 significance level, as per ANOVA and DMRT.

**Table 2:** Impact of Hg on composition of pigment in *Lens culinaris* L. variety NDL-2, with or without SNP and GR24.

Treatment	Chlorophyll a (mg g^−1^ FW)	Chlorophyll b (mg g^−1^ FW)	Total Chlorophyll (a + b) (mg g^−1^ FW)	Carotenoids (mg g^−1^ FW)
Control	1.61 ± 0.01 ^b^	0.76 ± 0.03 ^a^	2.37 ± 0.03 ^a^	0.49 ± 0.01 ^b^
Hg	1.17 ± 0.08 ^c^	0.22 ± 0.01 ^d^	1.39 ± 0.01 ^c^	0.43 ± 0.02 ^b^
SNP	1.72 ± 0.03 ^a^	0.77 ± 0.01 ^a^	2.49 ± 0.01 ^a^	0.53 ± 0.03 ^a^
GR24	1.8 ± 0.07 ^a^	0.82 ± 0.02 ^a^	2.62 ± 0.04 ^a^	0.56 ± 0.02 ^a^
SNP + GR24	1.86 ± 0.04 ^a^	0.86 ± 0.03 ^a^	2.72 ± 0.02 ^a^	0.58 ± 0.01 ^a^
Hg + SNP	1.2 ± 0.07 ^b^	0.48 ± 0.01 ^c^	1.68 ± 0.02 ^c^	0.48 ± 0.04 ^b^
Hg + GR24	1.23 ± 0.11 ^b^	0.63 ± 0.01 ^c^	1.86 ± 0.03 ^b^	0.51 ± 0.01 ^a^
Hg + SNP + GR24	1.45 ± 0.03 ^b^	0.71 ± 0.01 ^a^	2.16 ± 0.04 ^b^	0.52 ± 0.01 ^a^

Data presented are mean ± sem (*n* = 3). Different letters reflect significant differences among treatments at a *p* < 0.05 significance level, as per ANOVA and DMRT.

**Table 3:** Impact of Hg on sugar, proline, glycine betaine and protein contents of *Lens culinaris* L. variety NDL-2, with or without SNP and GR24.

Treatment	Sugar (mg g^−1^ DW)	Proline (µM g^−1^ DW)	Glycine Betaine (µg g^−1^ DW)	Protein (mg g^−1^ FW)
Control	4.15 ± 0.03 ^c^	15.25 ± 0.16 ^d^	2.33 ± 0.16 ^c^	15.29 ± 0.10 ^a^
Hg	3.06 ± 0.04 ^c^	19.32 ± 0.09 ^d^	6.17 ± 0.08 ^a^	9.34 ± 0.11 ^c^
SNP	4.89 ± 0.07 ^b^	25.42 ± 0.36 ^c^	2.77 ± 0.48 ^c^	13.66 ± 0.20 ^b^
GR24	5.13 ± 0.04 ^b^	27.33 ± 0.22 ^c^	3.13 ± 0.11 ^b^	14.91 ± 0.05 ^b^
SNP + GR24	6.75 ± 0.16 ^a^	34.69 ± 0.19 ^b^	3.67 ± 0.10 ^b^	17.14 ± 0.03 ^a^
Hg + SNP	5.43 ± 0.17 ^b^	37.38 ± 0.29 ^b^	6.5 ± 0.32 ^a^	12.95 ± 0.06 ^b^
Hg + GR24	5.94 ± 0.05 ^b^	40.29 ± 0.27 ^a^	6.9 ± 0.21 ^a^	14.17 ± 0.08 ^b^
Hg + SNP + GR24	7.16 ± 0.04 ^a^	48.11 ± 0.13 ^a^	7.8 ± 0.37 ^a^	16.64 ± 0.25 ^a^

Data presented are mean ± sem (*n* = 3). Different letters on the values show variation among treatments at the *p* < 0.05 significance level, as per ANOVA and DMRT.

**Table 4:** Accumulation of Hg in different parts of lentil seedlings, with and without SNP and GR24.

Treatment	Hg Content (µg g^−1^ DW)		Concentration Index (CI)		Translocation Factor (TF)
	Root	Shoot	Root	Shoot	
Control	0.16 ± 0.01 ^c^	0.13 ± 0.004 ^c^	3.06	3.06	0.81
Hg	9.63 ± 0.04 ^a^	7.29 ± 0.07 ^a^	60.19	56.08	0.76
SNP	5.26 ± 0.01 ^b^	4.19 ± 0.06 ^b^	32.88	32.23	0.79
GR24	4.95 ± 0.03 ^b^	3.82 ± 0.19 ^b^	30.94	29.38	0.77
SNP + GR24	4.12 ± 0.09 ^b^	3.11 ± 0.07 ^b^	25.75	23.92	0.75
Hg + SNP	6.03 ± 0.03 ^a^	5.27 ± 0.05 ^a^	37.69	40.54	0.87
Hg + GR24	6.82 ± 0.09 ^a^	6.11 ± 0.01 ^a^	42.63	47	0.89
Hg + SNP + GR24	5.95 ± 0.04 ^a^	5.08 ± 0.07 ^a^	37.19	39.08	0.85

Data presented are mean ± sem (*n* = 3). Different letters in the data reflect significant variation among treatments at a *p* < 0.05 significance level, according to ANOVA and DMRT.

**Table 5:** Effects of different treatments on uptake of minerals by lentil roots and shoots.

Lentil Plant	Minerals (µg g^−1^ DW)	Treatment							
		Control	Hg	SNP	GR24	SNP + GR24	Hg + SNP	Hg + GR24	Hg + SNP + GR24
Root	N	827 ± 1.41 ^b^	524 ± 2.16 ^c^	884 ± 2.16 ^a^	903 ± 1.63 ^a^	933 ± 2.16 ^a^	653 ± 4.97 ^a^	567 ± 1.41 ^a^	689 ± 2.12 ^a^
	P	384 ± 1.78 ^c^	121 ± 0.82 ^d^	420 ± 0.71 ^b^	424 ± 3.34 ^b^	452 ± 2.94 ^b^	346 ± 3.94 ^b^	321 ± 1.5 ^b^	366 ± 3.54 ^b^
	K	1324 ± 3.63 ^a^	705 ± 1.87 ^b^	1365 ± 15.20 ^a^	1406 ± 3.56 ^a^	1416 ± 5.72 ^a^	1219 ± 0.41 ^a^	1172 ± 2.48 ^a^	1294 ± 3.56 ^a^
	Ca	203 ± 1.77 ^b^	62 ± 0.41 ^d^	218 ± 0.41 ^c^	222 ± 4.32 ^b^	229 ± 4.49 ^b^	158 ± 2.55 ^c^	142 ± 0.82 ^c^	164 ± 1.47 ^c^
	Mg	264 ± 1.08 ^b^	83 ± 1.08 ^d^	269 ± 1.1 ^c^	274 ± 0.41 ^b^	281 ± 2.16 ^b^	198± 0.71 ^c^	155 ± 1.22 ^c^	203 ± 2.04 ^b^
Shoot	N	731 ± 0.82 ^a^	421 ± 1.78 ^b^	778 ± 3.19 ^a^	804 ± 2.86 ^a^	833 ± 2.55 ^a^	689 ± 6.72 ^a^	632 ± 1.1 ^a^	703 ± 1.08 ^a^
	P	285 ± 2.27 ^b^	93 ± 0.41 ^d^	289 ± 5.21 ^c^	296 ± 0.82 ^b^	308 ± 5.72 ^b^	252 ± 1.47 ^b^	248 ± 1.08 ^b^	268 ± 1.41 ^b^
	K	906 ± 1.1 ^a^	432 ± 1.47 ^b^	873 ± 1.08 ^a^	996 ± 1.77 ^a^	1013 ± 5.09 ^a^	862 ± 4.97 ^a^	721 ± 0.41 ^a^	675 ± 4.32 ^a^
	Ca	175 ± 0.41 ^c^	54 ± 1.41 ^d^	186 ± 0.82 ^d^	194 ± 3.89 ^c^	205 ± 1.47 ^b^	153 ± 1.78 ^c^	137 ± 3.54 ^c^	145 ± 3.54 ^c^
	Mg	214 ± 0.71 ^b^	63 ± 0.40 ^d^	222 ± 1.87 ^c^	237 ± 1.47 ^b^	244 ± 2.04 ^b^	172 ± 1.77 ^c^	139 ± 2.48 ^c^	181 ± 0.41 ^c^

Data are presented are mean ± sem (*n* = 3). Different letters in the data show significant differences among treatments at a *p* < 0.05 significance level, according to ANOVA and DMRT.

## Data Availability

The datasets generated during and/or analyzed during the current study are available from the corresponding author upon reasonable request.

## References

[1] Rai K.K., Pandey N., Meena R.P., Rai S.P. (2021). Biotechnological strategies for enhancing heavy metal tolerance in neglected and underutilized legume crops: A comprehensive review. Ecotoxicol. Environ. Saf..

[2] Chaki M., Begara-Morales J.C., Barroso J.B. (2020). Oxidative stress in plants. Antioxidants.

[3] Riyazuddin R., Nisha N., Ejaz B., Khan M.I.R., Kumar M., Ramteke P.W., Gupta R. (2021). A Comprehensive Review on the Heavy Metal Toxicity and Sequestration in Plants. Biomolecules.

[4] Du J., Guo Z., Li R., Ali A., Guo D., Lahori A.H., Wang P., Liu X., Wang X., Zhang Z. (2020). Screening of Chinese mustard (*Brassica juncea* L.) cultivars for the phytoremediation of Cd and Zn based on the plant physiological mechanisms. Environ. Pollut..

[5] Ganguly R., Sarkar A., Acharya K., Keswani C., Minkina T., Mandzhieva S., Sushkova S., Chakraborty N. (2022). The role of no in the amelioration of heavy metal stress in plants by individual application or in combination with phytohormones, especially auxin. Sustainability.

[6] Zhao S., Terada A., Nakamura K., Nakashima M., Komai T., Riya S., Hosomi M., Hou H. (2021). Significance of soil moisture on temperature dependence of Hg emission. J. Environ. Manag..

[7] Chou C.P., Chang T.C., Chiu C.H., Hsi H.C. (2018). Mercury speciation and mass distribution in cement production process of Taiwan. Aerosol Air Qual. Res..

[8] Hao R., Yang F., Mao X., Mao Y., Zhao Y., Lu Y. (2018). Emission factors of mercury and particulate matters, and in situ control of mercury during the co-combustion of anthracite and dried sawdust sludge. Fuel.

[9] Aysin F., Karaman A., Yilmaz A., Aksakal O., Gezgincioglu E., Kohnehshahri S.M. (2020). Exogenous cysteine alleviates mercury stress by promoting antioxidant defence in maize (*Zea mays* L.) seedlings. Turkish J. Agric. For..

[10] Han Y., Ni Z., Li S., Qu M., Tang F., Mo R., Ye C., Liu Y. (2018). Distribution, relationship, and risk assessment of toxic heavy metals in walnuts and growth soil. Environ. Sci. Pollut. Res..

[11] Campos J., Esbrí J., Madrid M., Naharro R., Peco J., García-Noguero E. (2018). Does mercury presence in soils promote their microbial activity? The Almadenejos case (Almadén mercury mining district, Spain). Chemosphere.

[12] Carvalho G.S., Oliveira J.R., Curi N., Schulze D.G., Marques J.J. (2019). Selenium and mercury in Brazilian Cerrado soils and their relationships with physical and chemical soil characteristics. Chemosphere.

[13] Mei L., Zhu Y., Zhang X., Zhou X., Zhong Z., Li H., Li Y., Li X., Daud M.K., Chen J. (2021). Mercury-induced phytotoxicity and responses in upland cotton (*Gossypium hirsutum* L.) seedlings. Plants.

[14] Shahid M., Khalid S., Bibi I., Bundschuh J., Niazi N.K., Dumat C. (2020). A critical review of mercury speciation, bioavailability, toxicity and detoxification in soil-plant environment: Ecotoxicology and health risk assessment. Sci. Total Environ..

[15] Jyothi N.R., Farook N.A.M. (2020). Mercury toxicity in public health. Heavy Met. Toxic. Public Health.

[16] Safari F., Akramian M., Salehi-Arjmand H., Khadivi A. (2019). Physiological and molecular mechanisms underlying salicylic acid-mitigated mercury toxicity in lemon balm (*Melissa officinalis* L). Ecotoxicol. Environ. Saf..

[17] Tamashiro H., Arakaki M., Akagi H., Futatsuka M., Roht L.H. (1985). Mortality and survival for minamata disease. Int. J. Epidemiol..

[18] Piscopo M., Tenore G.C., Notariale R., Maresca V., Maisto M., De Ruberto F., Heydari M., Sorbo S., Basile A. (2020). Antimicrobial and antioxidant activity of proteins from *Feijoa sellowiana* Berg. fruit before and after in vitro gastrointestinal digestion. Nat. Prod. Res..

[19] Tortora F., Notariale R., Maresca V., Good K.V., Sorbo S., Basile A., Piscopo M., Manna C. (2019). Phenol-rich *Feijoa sellowiana* (pineapple guava) extracts protect human red blood cells from mercury-induced cellular toxicity. Antioxidants.

[20] Henriques M.C., Loureiro S., Fardilha M., Herdeiro M.T. (2019). Exposure to mercury and human reproductive health: A systematic review. Rep. Toxicol..

[21] Sarkar A., Chakraborty N., Acharya K. (2021). Unraveling the role of nitric oxide in regulation of defense responses in chilli against *Alternaria* leaf spot disease. Physiol. Mol. Plant Pathol..

[22] Fujita M., Hasanuzzaman M. (2022). Approaches to enhancing antioxidant defense in plants. Antioxidants.

[23] Li R., Wu H., Ding J., Fu W., Gan L., Li Y. (2017). Mercury pollution in vegetables, grains and soils from areas surrounding coal-fired power plants. Sci. Rep..

[24] Zhang T., Lu Q., Su C., Yang Y., Hu D., Xu Q. (2017). Mercury induced oxidative stress, DNA damage, and the activation of antioxidative system and Hsp 70 induction in duckweed (*Lemna minor*). Ecotoxicol. Environ. Saf..

[25] Sun H., Feng F., Liu J., Zhao Q. (2018). Nitric oxide affects rice root growth by regulating auxin transport under nitrate supply. Front. Plant Sci..

[26] Zhang H., Huo S., Yeager K.M., Xi B., Zhang J., He Z. (2018). Accumulation of arsenic, mercury and heavy metals in lacustrine sediment in relation to eutrophication: Impacts of sources and climate change. Ecol. Indic..

[27] Ahmad P., Ahanger M.A., Alyemeni M.N., Wijaya L., Alam P. (2018). Exogenous application of nitric oxide modulates osmolytes metabolism, antioxidants, enzymes of ascorbate-glutathione cycle and promotes growth under cadmium stress in tomato. Protoplasma.

[28] Azevedo R., Rodriguez E., Mendes R.J., Mariz-Ponte N., Sario S., Lopes J.C., de Oliveira J.M.P.F., Santos C. (2018). Inorganic Hg toxicity in plants: A comparison of different genotoxic parameters. Plant Physiol. Biochem..

[29] Paul A., Sarkar A., Acharya K., Chakraborty N. (2022). Fungal elicitor-mediated induction of innate immunity in Catharanthus roseus against leaf blight disease caused by *Alternaria alternata*. J. Plant Growth Regul..

[30] Asgher M., Per T.S., Masood A., Fatma M., Freschi L., Corpas F.J., Khan N.A. (2017). Nitric Oxide Signaling and Its Crosstalk with Other Plant Growth Regulators in Plant Responses to Abiotic Stress. Environ. Sci. Pollut. Res. Int..

[31] Roychoudhury A., Ghosh S., Paul S., Mazumdar S., Das G., Das S. (2016). Pre-treatment of seeds with salicylic acid attenuates cadmium chloride-induced oxidative damages in the seedlings of mungbean (*Vigna radiata* L. Wilczek). Acta Physiol. Plant.

[32] Keishama M., Jaina P., Singha N., Toerneb C.V., Bhatla S.C., Lindermayr C. (2019). Deciphering the nitric oxide, cyanide and iron-mediated actions of sodium nitroprusside in cotyledons of salt stressed sunflower seedlings. Nitric Oxide.

[33] Banerjee A., Roychoudhury A. (2018). Strigolactones: Multi-level regulation of biosynthesis and diverse responses in plant abiotic stresses. Acta Physiol. Plant.

[34] Jamil M., Kountche B.A., Haider I., Wang J.Y., Aldossary F., Zarban R.A., Jia K.P., Yonli D., Shahul Hameed U.F., Takahashi I. (2019). Methylation at the c-3′ in d-ring of strigolactone analogs reduces biological activity in root parasitic plants and rice. Front Plant Sci..

[35] Sun H., Tao J., Liu S., Huang S., Chen S., Xie X., Yoneyama K., Zhang Y., Xu G. (2014). Strigolactones are involved in phosphate and nitrate-deficiency induced root development and auxin transport in rice. J. Exp. Bot..

[36] Rehman N.U., Ali M., Ahmad M.Z., Liang G., Zhao J. (2018). Strigolactones promote rhizobia interaction and increase nodulation in soybean (*Glycine max*). Microb. Pathog..

[37] Mostofa M.G., Li W., Nguyen K.H., Fujita M., Tran L.P. (2018). Strigolactones in plant adaptation to abiotic stresses: An emerging avenue of plant research. Plant Cell Environ..

[38] Chelladurai V., Erkinbaev C., Manickavasagan A., Thirunathan P. (2020). Lentils. Pulses.

[39] Kaur C., Sharma S., Hasan M.R., Pareek A., Singla-Pareek S.L., Sopory S.K. (2017). Characteristic variations and similarities in biochemical, molecular, and functional properties of glyoxalases across prokaryotes and eukaryotes. Int. J. Mol. Sci..

[40] Iqbal M.Z., Shafiq M., Athar M. (2014). Phytotoxic effects of mercury on seed germination and seedling growth of *Albizia lebbeck* L. Benth. (Leguminosae). Adv. Environ. Res..

[41] Rizvi A., Zaidi A., Ameen F., Ahmed B., AlKahtani M.D., Khan M.S. (2020). Heavy metal induced stress on wheat: Phytotoxicity and microbiological management. RSC Adv..

[42] Cui W., Fang P., Zhu K., Mao Y., Gao C., Xie Y., Wang J., Shen W. (2014). Hydrogen-rich water confers plant tolerance to mercury toxicity in alfalfa seedlings. Ecotoxicol. Environ. Saf..

[43] Magistrali P.R., Borges E.E.D.E., Oliveira J.A., Faria J.M.R., Ataide G.D., Nascimento J.F. (2019). Mercury affects aquaporins activity and germination of the embryonic axis of *Schizolobium parahyba* (vell. Blake (fabaceae). Rev. Arvore.

[44] Kim Y.X., Stumpf B., Sung J., Lee S.J. (2018). The relationship between turgor pressure change and cell hydraulics of midrib parenchyma cells in the leaves of *Zea mays*. Cells.

[45] Basalah M.O., Ali H.M., Al-Whaibi M.H., Siddiqui M.H., Sakran A.M., Al Sahli A.A. (2013). Oxide and Salicylic Acid Mitigate Cadmium Stress in. Wheat Seedlings. J. Pure. Appl. Microbiol..

[46] Ahmad P., Abdel Latef A.A., Hashem A., Abd-Allah E.F., Gucel S., Tran L.S.P. (2016). Nitric oxide mitigates salt stress by regulating levels of osmolytes and antioxidant enzymes in chickpea. Front. Plant Sci..

[47] Kolbert Z. (2016). Implication of nitric oxide (no) in excess element-induced morphogenic responses of the root system. Plant Physiol. Biochem..

[48] Bin-Jumah M., Abdel-Fattah A.F.M., Saied E.M., El-Seedi H.R., Abdel-Daim M.M. (2021). Acrylamide-induced peripheral neuropathy: Manifestations, mechanisms, and potential treatment modalities. Environ. Sci. Pollut. Res..

[49] Kharbech O., Sakouhi L., Ben Massoud M., Mur L.A., Corpas F.J., Djebali W., Chaoui A. (2020). Nitric oxide and hydrogen sulfide protect plasma membrane integrity and mitigate chromium-induced methylglyoxal toxicity in maize seedlings. Plant Physiol. Biochem..

[50] Gaber A., Alsanie W.F., Kumar D.N., Refat M.S., Saied E.M. (2020). Novel papaverine metal complexes with potential anticancer activities. Molecules.

[51] Kharbech O., Houmani H., Chaoui A., Corpas F.J. (2017). Alleviation of Cr(VI)-induced oxidative stress in maize (*Zea mays* L.) seedlings by NO and H2S donors through differential organ-dependent regulation of ROS and NADPH-recycling metabolisms. J. Plant Physiol..

[52] Rizwan M., Mostofa M.G., Mz A. (2018). Nitric oxide induces rice tolerance to excessive nickel by regulating nickel uptake, reactive oxygen species detoxification and defense-related gene expression. Chemosphere.

[53] Demecsova L., Bočova B., Zelinova V., Tamas L. (2019). Enhanced nitric oxide generation mitigates cadmium toxicity via superoxide scavenging leading to the formation of peroxynitrite in barley root tip. J. Plant Physiol..

[54] Kolbert Z. (2019). Strigolactone-nitric oxide interplay in plants: The story has just begun. Physiol. Plant.

[55] Ahmad M.Z., Rehman N.U., Yu S., Zhou Y., Haq B.U., Wang J., Li P., Zeng Z., Zhao J. (2020). Gm MAX2-D14 and -KAI interaction mediated SL and KAR signaling play essential roles in soybean root nodulation. Plant J..

[56] Teixeira D.C., Lacerda L.D., Silva-Filho E.V. (2018). Foliar mercury content from tropical trees and its correlation with physiological parameters in situ. Environ. Pollut..

[57] Sharma A., Soares C., Sousa B., Martins M., Kumar V., Shahzad B., Sidhu G.P., Bali A.S., Asgher M., Bhardwaj R. (2019). Nitric oxide-mediated regulation of oxidative stress in plants under metal stress: A review on molecular and biochemical aspects. Physiol. Plant.

[58] Yang J., Li G., Bishopp A., Heenatigala P.P.M., Hu S., Chen Y., Wu Z., Kumar S., Duan P., Yao L. (2018). A Comparison of growth on mercuric chloride for three lemnaceae species reveals differences in growth dynamics that effect their suitability for use in either monitoring or remediating ecosystems contaminated with mercury. Front. Chem..

[59] Min Z., Li R., Chen L., Zhang Y., Li Z., Liu M., Ju Y., Fang Y. (2018). Alleviation of drought stress in grapevine by foliar-applied strigolactones. Plant Physiol. Biochem..

[60] Ben Massoud M., Sakouhi L., Karmous K. (2018). Protective role of exogenous phytohormones on redox status in pea seedlings under copper stress. J. Plant Physiol..

[61] Cabrita M.T., Duarte B., Cesário R., Mendes R., Hintelmann H., Eckey K., Dimock B., Caçador I., Canário J. (2019). Mercury mobility and effects in the salt-marsh plant Halimione portulacoides: Uptake, transport, and toxicity and tolerance mechanisms. Sci. Total Environ..

[62] Kaya C., Ashraf M. (2015). Exogenous application of nitric oxide promotes growth and oxidative defense system in highly boron stressed tomato plants bearing fruit. Sci. Hort..

[63] Asada K. (1992). Ascorbate peroxidase—A hydrogen peroxide-scavenging enzyme in plants. Physiol. Plantarum..

[64] Foyer C.H., Noctor G. (2005). Redox homeostasis and antioxidant signaling: A metabolic interface between stress perception and physiological responses. Plant Cell.

[65] Reddy P.S., Jogeswar G., Rasineni G.K., Maheswari M., Reddy A.R., Varshney R.K. (2015). Proline over-accumulation alleviates salt stress and protects photosynthetic and antioxidant enzyme activities in transgenic sorghum. Moench. Plant Physiol. Biochem..

[66] Choudhary S., Wani K.I., Naeem M., Khan M.M.A., Aftab T. (2022). Cellular responses, osmotic adjustments, and role of osmolytes in providing salt stress resilience in higher plants: Polyamines and nitric oxide crosstalk. J. Plant Growth Regul..

[67] Dumanovic J., Nepovimova E., Natic M., Kuca K., Jacevic V. (2021). The significance of reactive oxygen species and antioxidant defense system in plants: A concise overview. Front. Plant Sci..

[68] Sankaranarayanan S., Jamshed M., Kumar A., Skori L., Scandola S., Wang T., Spiegel D., Samuel M.A. (2017). Glyoxalase goes green: The expanding roles of glyoxalase in plants. Int. J. Mol. Sci..

[69] Suhartono E., Triawanti T., Setyo Leksono A., Sasmito Djati M. (2014). The role of cadmium in proteinsglycation by glucose: Formation of methylglyoxal and hydrogenper- oxide invitro. J. Med. Bioeng..

[70] Chowardhara B., Borgohain P., Saha B., Awasthi J.P., Panda S.K. (2020). Differential oxidative stress responses in *Brassica juncea* (L.) Czern and Coss cultivars induced by cadmium at germination and early seedling stage. Acta Physiol. Plant.

[71] Xu L.L., Fan Z.Y., Dong Y.J., Kong J., Bai X.Y. (2015). Effects of exogenous salicylic acid and nitric oxide on physiological characteristics of two peanut cultivars under cadmium stress. Biol. Plant..

[72] Nahar K., Rhaman M.S., Parvin K., Bardhan K., Marques D.N., García-Caparrós P., Hasanuzzaman M. (2022). Arsenic-induced oxidative stress and antioxidant defense in plants. Stresses.

[73] Pirzadah T.B., Malik B., Tahir I., Irfan Q.M., Rehman R.U. (2018). Characterization of mercury-induced stress biomarkers in Fagopyrum tataricum plants. Inter. J. Phytoremed..

[74] Ben Massoud M., Kharbech O., Mahjoubi Y., Chaoui A., Wingler A. (2022). Effect of exogenous treatment with nitric oxide (no) on redox homeostasis in barley seedlings (*hordeum vulgare* l.) under copper stress. J. Soil Sci. Plant Nutr..

[75] Kapoor R.T., Hefft D.I., Ahmad A. (2023). Nitric oxide and spermidine alleviate arsenic-incited oxidative damage in *Cicer arietinum* by modulating glyoxalase and antioxidant defense system. Funct. Plant Biol..

[76] Alsahli A.A., Bhat J.A., Alyemeni M.N. (2021). Hydrogen sulphide mitigates arsenic induced toxicity in pea plants by regulating osmoregulating osmoregulation, antioxidant defense system, ascorbate glutathione cycle and glyoxylase system. J. Plant Growth Regul..

[77] Lichtenthaler H.K. (1987). Chlorophylls and carotenoids pigments of photosynthetic membranes. Methods Enzymol..

[78] Hedge J.E., Hofreiter B.T., Whistler R.L., Wolfrom M.L. (1962). Estimation of carbohydrate. Methods in Carbohydrate Chemistry.

[79] Bates L.S., Waldren R.P., Teare I.D. (1973). Rapid determination of proline for water-stress studies. Plant Soil.

[80] Grieve C.M., Grattan S.R. (1983). Rapid assay for the determination of water soluble quaternary ammonium compounds. Plant Soil.

[81] Lowry O.H., Rosebrough N.J., Fan A.L., Randall R.I. (1951). Protein measurement with the folin phenol reagent. J. Biol. Chem..

[82] Dionisio-Sese M.L., Tobita S. (1998). Antioxidant responses of rice seedlings to salinity stress. Plant Sci..

[83] Velikova V., Yordanov I., Edreva A. (2000). Oxidative stress and some antioxidant systems in acid rain-treated bean plants: Protective role of exogenous polyamines. Plant Sci..

[84] Yang L., Tian D., Todd C.D., Luo Y., Hu X. (2013). Comparative proteome analyses reveal that nitric oxide is an important signal molecule in the response of rice to aluminum toxicity. J. Proteome Res..

[85] Zhou W., Leul M. (1998). Uniconazole-induced alleviation of freezing injury in relation to changes in hormonal balance, enzyme activities and lipid peroxidation in winter rape. Plant Growth Regul..

[86] Doderer A., Kokkelink I., Vanderween S., Valk B., Schrom A.W., Douma A.C. (1992). Purification and characterization of two lipoxygenase isoenzymes from germinating barley. Biochim. Biophys. Acta.

[87] Beyer W.F., Fridovich I. (1987). Assaying for superoxide dismutase activity some large consequences of minor changes in conditions. Anal. Biochem..

[88] Cakmak I., Marschner H. (1992). Magnesium deficiency and high light intensity enhance activities of superoxide dismutase, ascorbate peroxidase and glutathione reductase in bean leaves. Plant Physiol..

[89] Nakano Y., Asada K. (1981). Hydrogen peroxide is scavenged by ascorbate specific peroxidase in spinach chloroplasts. Plant Cell Physiol..

[90] Foster J.S., Hess J.L. (1980). Responses of superoxide dismutase and glutathione reductase activities in cotton leaf tissue exposed to an atmosphere enriched in oxygen. Plant Physiol..

[91] Hasanuzzaman M., Hossain M.A., Fujita M. (2011). Nitric oxide modulates antioxidant defense and the methylglyoxal detoxification system and reduces salinity-induced damage of wheat seedlings. Plant Biotechnol. Rep..

[92] Kaya C., Higgs D., Ashraf M., Alyemeni M.N., Ahmad P. (2020). Integrative roles of nitric oxide and hydrogen sulfide in melatonin-induced tolerance of pepper (*Capsicum annuum* L.) plants to iron deficiency and salt stress alone or in combination. Physiol. Plant..

[93] Wild R., Ooi L., Srikanth V., Münch G. (2012). A quick, convenient and economical method for the reliable determination of methylglyoxal in millimolar concentrations: The N-acetyl-L-cysteine assay. Anal. Bioanal. Chem..

[94] Mukherjee S.P., Choudhuri M.A. (1983). Implications of water stressinduced changes in the levels of endogenous ascorbic acid and hydrogen peroxide in *Vigna* seedlings. Physiol. Plant.

[95] Ellman G.L. (1959). Tissue sulfhydryl groups. Arch. Biochem. Biophys..

